# Turning over New Leaves

**Published:** 1888-12-22

**Authors:** 


### TURNING OVER NEW LEAVES.

(Books for Review should be sent to The Editor. The Lodge, Porchester Square, W.)

### The "Universal Review."

THE struggle for existence is severe for a new journal. Before it appears hardly anybody sees a necessity for it; it has to prove itself, if not better, at least different from any other before it is accepted by the public. Though the "Universal Review" has not yet completed a year of existence, we may venture to congratulate Mr. Harry Quilter on a complete success. There is nothing in the wide world of English magazines exactly like it. Not only does it give the widest possible scope to English writers of all shades of opinion, but the studies of French art, so admirably illustrated, which it generally contains, open up new worlds to our insular imaginations. This month, Mr. Quilter gives us an article by Louis Tourcand on Willette, interspersed with exquisite sketches by that artist, full of that humour which makes one very sad. The genius, the subtlety, the infinite variety of meaning one can find in them, the direct bearing on human life, shown in the allegorical history of the white-robed Pierrot, are altogether unique. Pierrot is an idealist—an inveterate idealist—who, disillusioned on every side, still dreams on. Always blessed by the best intentions, he can see nothing but good till evil comes upon him; so innocent is he and so shortsighted that, among other adventures, we find him dancing arm-in-arm with undertakers and mutes at the advent of the cholera.

It must be admitted that there is a good deal that is gloomy in the December number of the "Universal." Anything more scathing in its wit than Mr. Traill's "Doom of the Muses" can hardly be conceived. It is terribly clever, uncomfortably true. In tragedy, according to Mr. Traill, we worship the tragedian, in comedy the chief actress's gowns. The votaries of music cannot decide what music is; history is buried under a mass of "original authorities," epic poetry is wholly forsaken, and the offerings laid on the altar of the lyric muse are either hopelessly fantastic or hopelessly incomprehensible. What the makers and lovers of "the ballade, with the final *e*," will say to Mr. Traill's ballade on the assonants to "spanks" we prefer not to conjecture.

We very much doubt if Mr. Traill would wholly appreciate the Rev. Frederick Rolfe's sestina on little Saint Hugh of Lincoln. We confess to thinking the sestina one of the most painfully artificial forms of artificial verse; but the beauty of the initial letters of each verse, picturing the story of Saint Hugh's martyrdom and canonisation, will satisfy those who like the poem least. It is certainly praiseworthy, but one turns from it with a sense of relief to Mr. Swinburne's swinging, breezy stanzas on "Æolus."

It required an artist like Mr. Hardy to make a story out of the incident on which he has built "A Tragedy of Two Ambitions," the drowning of a drunken and incorrigible vagabond father in sight of his two sons, and their fatal delay in trying to rescue him, caused by the consciousness that he will destroy beyond retrieving the honourable positions they have so hardly obtained. It is painful reading; one is so much in sympathy with the sons that the feeling that one ought to disapprove of their conduct jars a little. Mr. Quilter's review of the year is a thoughtful piece of work, carefully moderate in tone. He will surprise most people, especially the sworn admirers of "Robert Elsmere," by estimating Lucas Malet's "Counsel of Perfection" as the most important work done in fiction during the year; but people who have studied the things that go to make literature will hesitate to contradict him. The "Universal Review" has a good record in its brief past, and bears good promise for the future.

### Plays for Children.*

THIS is a prettily-bound little book, containing four short plays written for children, to act during their Christmas holidays. The first piece is on the subject of the Sleeping Beauty, and is written in rhyming lines. There are two scenes, which are easily arranged, and about ten actors are required. The scene where the inhabitants of the palace, from the king down to the cook, are overcome by sleep, is cleverly written, and should make an effective bit of acting. The other three plays are founded on the well-known stories of "Jack and the Beanstalk," "The White Cat," and "Snowdrop and the Seven Dwarfs." It is a pity that giants, and dwarfs, and beanstalks, rather tax the powers of the amateur theatrical manager; difficulties, however, which do not intrude in the play of the "White Cat." This last certainly seems to us the best play for acting: each character is strongly drawn, with just the touch of exaggeration necessary for stage purposes, and the plot is more original and the dialogue amusing. Each play is sold separately for sixpence, and practical directions are given with regard to costume, etc., no effort having been spared to make these plays popular with little actors.

* "Terra Cotta Plays." By C. M. Prevost. (Smith and Innes.)

